# Prognostic significance of carbohydrate antigen 125 in stage D heart failure

**DOI:** 10.1186/s12872-023-03139-5

**Published:** 2023-02-25

**Authors:** Ji Zhang, Wenhua Li, Jianqiang Xiao, Jie Hui, Yi Li

**Affiliations:** 1 Department of Cardiology, Wujin Hospital Affiliated With Jiangsu University, The Wujin Clinical College of Xuzhou Medical University, No. 2, Yongning Road, Changzhou, Jiangsu Province China; 2 Department of Cardiology, The First Affiliated Hospital of Soochow University, Suzhou, China; 3 School of Math and Statistics, Nanjing Audit University, Nanjing, China

**Keywords:** Carbohydrate antigen 125, Prognosis, Stage D heart failure

## Abstract

**Background:**

The predictive value of carbohydrate antigen 125 (CA125) has not been examined in stage D heart failure (HF) patients, regardless of left ventricular ejection fraction (LVEF). We sought to quantify the prognostic usefulness in predicting death and HF readmission in this cohort.

**Methods:**

According to CA125 levels above and below the median (65.7 U/ml), 176 stage D HF patients including more than half (50.6%) had LVEF > 40% were divided into 2 groups.

**Results:**

A total of 106 (60.2%) deaths and 102 deaths due to the cardiovascular disease were identified. All-cause death/HF readmission and MACE occurred in 157 patients (89.2%) during 18 months (16–20) of follow-up. By the Kaplan–Meier method, subjects with CA125 ≥ 65.7 U/ml exhibited higher 1-year mortality rate (59.3% vs. 31.0%, *P* < 0.001) and 1-year death/HF rehospitalization rate (94.2% vs. 80.6%, *P* < 0.001). In univariate Cox analysis, CA125 (categorized) was a significant prognostic factor for all-cause death, cardiovascular mortality, death/HF readmission and MACE. Based on multivariate Cox analysis, elevated CA125 was still significant for all-cause death, cardiovascular mortality, death/HF readmission and MACE.

**Conclusions:**

In stage D HF patients, elevated CA125 levels were highly predictive of all-cause death, cardiovascular mortality, all-cause death/HF readmission and MACE, which can be used for better risk stratification.

## Introduction

The heart failure (HF) syndrome affects approximately more than 23 million people worldwide [1, 2]. As medical and surgical therapy have extended life, patients are increasingly living with stage D HF. It was reported that the rate of progression to stage D each year among outpatients with stage C heart failure with reduced ejection fraction (HFrEF) was 4.5% and it was reported that the rate of progression to stage D each year among outpatients with stage C HFrEF was 4.5% and 5% surviving with end-stage disease among all HF patients [3].

Given the highly variable clinical course of stage D HF, accurate prognostication is difficult. Natriuretic peptides (NPs) and other biomarkers for myocardial remodeling (e.g. galectin-3, ST-2, etc.) have not been shown to be a robust independent prognostic factor in subjects with stage D HF [4]. As objective measures of functional capacity, cardiopulmonary exercise test or 6-min walk are usually difficult to achieve in older frail stage D patients. Multimarker HF survival models such as the Seattle Heart Failure Score and Heart Failure Survival Score was derived from younger and mild-to-moderate HF populations, resulting in poor prognostic performance in stage D HF [5, 6].

As a widely used biomarker for monitoring ovarian cancer [7], carbohydrate antigen 125 (CA125) is indicative of a HF severity surrogate [8], and correlates with adverse events in acute HF patients [9, 10]. Previous study has found a relationship between elevated CA125 and higher death or heart transplant rate in 88 severe advanced HF patients with low left ventricular ejection fraction (LVEF [24.5 ± 11.2%]) referred for heart transplantation (HT) [11]. Our study aimed to evaluate CA125's prognostic utility in a large group of stage D HF patients regardless of LVEF.

## Methods

### Study population and design

This was a prospective, observational cohort study from a single center that included 176 stage D HF patients with symptoms on minimal exertion or at rest (advanced New York Heart Association [NYHA] class III or IV) despite appropriate conventional therapy for HF [12] consecutively admitted to the cardiology ward from February 2019 to May 2021. Exclusion criteria were: history of HF less than 3 months, treatment with intravenous vasodilators or inotropic agents within 48 h prior to enrollment, N-terminal pro-B-type natriuretic peptide (NTproBNP) < 125 pg/ml, dyspnea not mainly due to HF, non-cardiac terminal illness (< 1 year life expectancy), recent acute coronary syndrome (within 3 months), cardiac resynchronization therapy within 3 months, cardiac catheter ablation within 30 days, cardiac revascularization within 30 days, history of malignancy within the past 2 years, active inflammation, serum creatinine > 250 µmol/L, severe anemia (haemoglobin < 60 g/L). Demographic information, vital signs, medications, medical history were collected, along with standard echocardiographic evaluation, laboratory results and 12-lead electrocardiogram at study enrollment.

The median follow-up was 18 months (16–20), with 1 patient lost to follow-up at 7 months. The primary endpoint was time to all-cause mortality. The secondary endpoints were composite of all-cause death or HF readmission, cardiovascular mortality and major adverse cardiovascular events (MACE), defined as a composite of death from cardiovascular causes, myocardial infarction, stroke, or heart failure. The local ethics committee approved this study, and all patients provided informed consent to their participation. All methods were performed in accordance with the Declaration of Helsinki.

### Biomarkers measurement

CA125 and NTproBNP serum levels were obtained at study enrollment using commercially available immunoassay kits (Elecsys CA125 II assay, Roche Diagnostics and Vitros Immunodiagnostic Products NT-proBNP Reagent Pack, Ortho-Clinical Diagnostics, respectively).

### Statistical analysis

Categorical variables were presented as frequencies and percentages, continuous variables summarized as median (interquartile range). According to CA125 levels above and below the median, we divided population into two groups and compared between-group baseline characteristics using Mann–Whitney test and chi-square. Median baseline levels of the entire population for CA125 and NTproBNP were 65.7 U/ml (25.3–129.5) and 4900 pg/ml (2910–8655), respectively. The cumulative rate of events and median survival time among CA125 categories were estimated and compared using the Kaplan–Meier method and log-rank test. The effects of various baseline variables on outcome were investigated by univariate Cox analyses. Continuous variables were grouped in clinically relevant classes to further characterize their impact on outcome. The impact of CA125 levels was assessed by multivariate Cox analyses. For multivariate Cox analyses, we retained significant factors for mortality in univariate Cox analysis (*P* < 0.05). Variables included in the final model were CA125 ≥ 65.7 U/ml, NTproBNP ≥ 4900 pg/ml, age ≥ 70 years, serum sodium < 135 mmol/L, presence of pleural effusion or ascites, recent admission ( at least 1 heart failure admission within 3 months) and treatment with digitalis. To test for interaction between CA125 and NTproBNP, an interaction term was added to multivariate Cox model for mortality. Calibration and discrimination of the Cox model for mortality were assessed by calibration curves and time-dependent ROC curves.

In all analyses, 2-sided *P*-value of < 0.05 was considered statistically significant. Statistical analysis was performed using SPSS 26.0 and R 4.2.1.

## Results

### Baseline characteristics

Of 176 subjects, 89 (50.6%) had LVEF > 40%. The sample was 77 (43.8%) female and the median age was 76 years (70–82). Baseline characteristics of study participants by CA125 categories are shown in Table 1. Groups were similar regarding demographic characteristics, HF etiology, medication, baseline heart rate, systolic blood pressure, LVEF, left ventricular diameter, serum creatinine, and serum sodium. Compared to subjects with CA125 below median, subjects with CA125 above median had higher NTproBNP and a higher proportion of pleural effusion or ascites, peripheral oedema, and pulmonary rales. 62 (35.2%) patients had normal CA125 (≤ 35 U/ml), while 114 (64.8%) had CA125 > 35 U/ml.

**Table 1:** Baseline characteristics stratified by CA125 categories

	CA125 < 65.7 U/ml (*n* = 88)	CA125 ≥ 65.7 U/ml (*n* = 88)	*P*-value
Demographic and medical history			
Age, years	76 (70–83)	76 (71–82)	0.965
Female, *n* (%)	38 (43.2)	39 (44.3)	0.879
Weight, kg	56.5 (50.0–68.4)	56.5 (47.0–65.0)	0.650
Hypertension, *n* (%)	45 (51.1)	40 (45.5)	0.451
Diabetes mellitus, *n* (%)	25 (28.4)	22 (25.0)	0.609
Atrial fibrillation, *n* (%)	41 (46.6)	52 (59.1)	0.097
Etiology (ischemic), *n* (%)	16 (18.2)	15 (17.0)	0.843
Pleural effusion or ascites, *n* (%)	25 (28.4)	72 (81.8)	< 0.001
Pulmonary rales, *n* (%)	37 (42.0)	63 (71.6)	< 0.001
Peripheral oedema, *n* (%)	31 (35.2)	47 (53.4)	0.015
Vital signs			
Heart rate, b.p.m	80 (70–90)	80 (70–92)	0.462
Systolic blood pressure, mmHg	120 (108–136)	121 (106–136)	0.653
Diastolic blood pressure, mmHg	73 (67–80)	74 (68–84)	0.655
Laboratory			
Haemoglobin (g/L)	127.5 (108.5–138.0)	124.0 (102.3–137.8)	0.559
Serum creatinine (umol/L)	109 (79–140)	96 (73–136)	0.218
Sodium (mmol/L)	139.3 (136.8–142.1)	138.8 (135.8–141.1)	0.181
NTproBNP (pg/ml)	4380 (2491–6235)	5670 (3735–10,950)	< 0.001
CA125 (U/ml)	25.4 (16.4–40.3)	127.9 (87.7–182.9)	< 0.001
Echocardiography			
LVEF (%)	44 (29–55)	40 (27–54)	0.444
LVEF < 50%, n (%)	59 (67.0)	58 (65.9)	0.873
Left ventricular diastolic diameter (mm)	58 (48–68)	59 (50–71)	0.353
Left ventricular systolic diameter (mm)	45 (36–58)	47 (37–57)	0.254
Left atrial diameter (mm)	49 (43–54)	51 (46–60)	0.004
Medical treatment			
Beta-blockers, *n* (%)	69 (78.4)	67 (76.1)	0.719
Spironolactone, *n* (%)	78 (88.6)	80 (90.9)	0.619
ACEI/ARB/ARNI, *n* (%)	52 (59.1)	49 (55.7)	0.647
Digitalis	29 (33.0)	32 (36.4)	0.635

CA125, carbohydrate antigen 125; NTproBNP, N-terminal pro-B-type natriuretic peptide; LVEF, left ventricular ejection fraction; ACEI, angiotensin converting enzyme inhibitors; ARB, angiotensin receptor blockers; ARNI, angiotensin receptor neprilysin inhibitor

### CA125 and clinical events

In total, 106 (60.2%) patients died (196 days [80–401]) (primary endpoint), and 102 deaths due to the cardiovascular disease were documented. Death or HF rehospitalizations occurred in 157 (89.2%) of 176 subjects. MACE occurred in 157 patients (89.2%) during follow-up. CA125 values in subjects experiencing death were significantly higher when compared with those who survived (82.3 U/ml [28.7–141.7] vs. 41.9 U/ml [22.2–90.5], *P* = 0.008). Table 2 shows median survival time according to CA125 values below and above median of 65.7 U/ml. CA125 above median correlated strongly with shorter median survival time.

**Table 2:** Median survival time and event rates per patient-year according to median CA125 level

	CA125 < 65.7 U/ml (*n* = 88)		CA125 ≥ 65.7 U/ml (*n* = 88)		*P*
	Median survival time, months (95% CI)	Events per patient-year, %	Median survival time, months (95% CI)	Events per patient-year, %	
All-cause mortality (*n* = 106)	17 (13–21)	45.5	8 (4–12)	100.6	< 0.001
All-cause mortality or heart failure rehospitalization (*n* = 157)	5 (4–6)	75.9	2 (1–3)	135.3	< 0.001

**Fig. 1:**
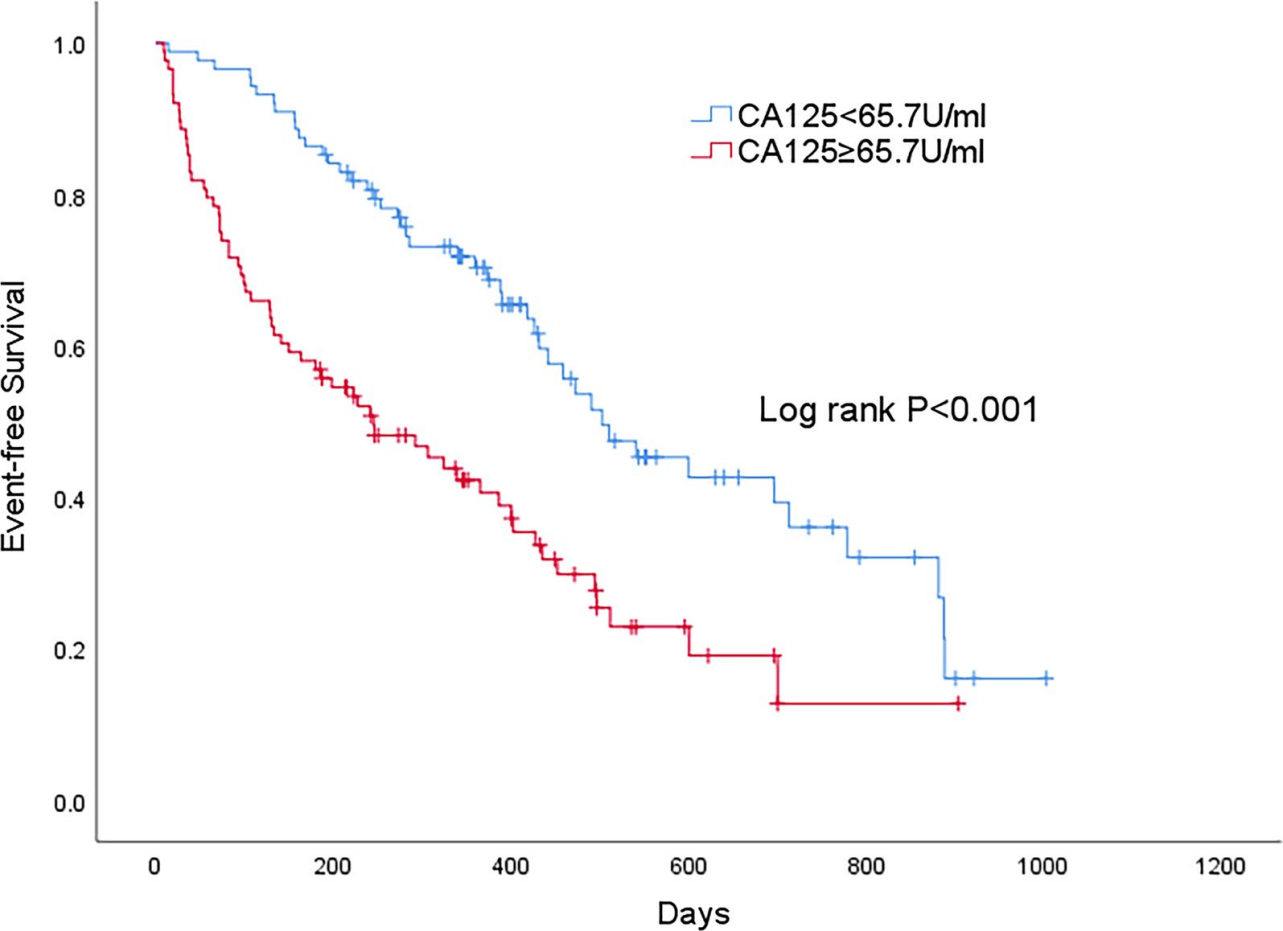
Kaplan–Meier estimates for all-cause mortality in patients with CA125 below and above median of 65.7 U/ml. 1-year mortality rate 59.3 versus 31.0%. Log-rank *P* < 0.001

**Fig. 2:**
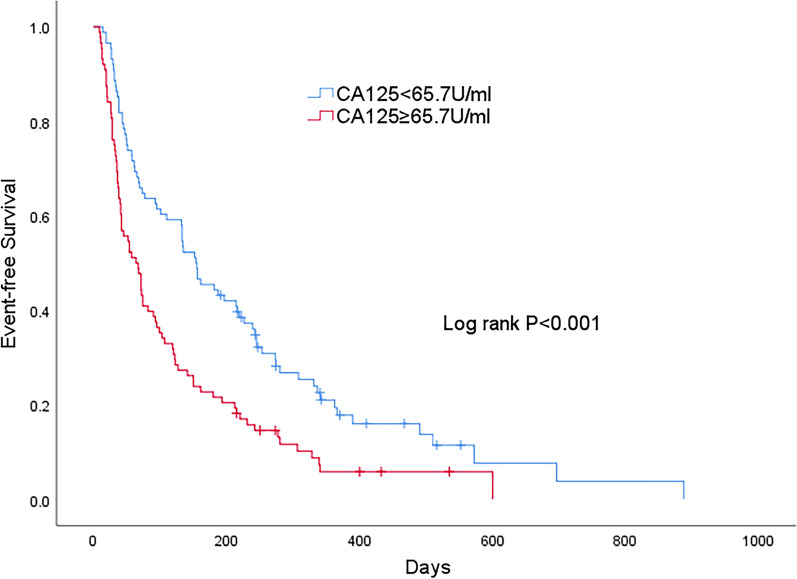
Kaplan–Meier estimates for the combined endpoint of death/heart failure rehospitalization in patients with CA125 below and above median of 65.7 U/ml. 1-year event (death or heart failure rehospitalization) rate 94.2 versus 80.6%. Log-rank *P* < 0.001

By the Kaplan–Meier method, subjects with CA125 ≥ 65.7 U/ml exhibited significantly higher 1-year mortality rate of 59.3% and shorter median survival time of 8 months (4–12), as compared with 31.0% and 17 months (13–21) in patients with CA125 < 65.7 U/ml (*P* < 0.001) as illustrated in Fig. 1. In subjects with CA125 ≥ 65.7 U/ml, the 1-year cumulative death/HF rehospitalization rate was 94.2% with a median survival time of 2 months (1–3), and in patients with CA125 < 65.7 U/ml 80.6% with a median survival time of 5 months (4–6) (*P* < 0.001) as shown in Fig. 2.

Tables 3 and 4 displayed the results of univariate Cox modeling. CA125 (categorized), NTproBNP (continuous or categorized), HF readmission within 3 months prior to enrollment (recent admission) were significant in the primary endpoint and combined endpoint of death/HF rehospitalization (*P* = 0.05 level). CA125 (categorized), NTproBNP (categorized) and recent admission were significant in the secondary endpoints of cardiovascular mortality and MACE. Serum sodium (continuous or categorized), age (categorized), heart rate (continuous), evidence of pleural effusion or ascites and treatment with digoxin reached significance on all-cause mortality, but was not significant in the secondary endpoint of death/HF rehospitalization. Serum sodium (categorized), evidence of pleural effusion or ascites, treatment with digoxin, ACEI/ARB/ARNI and spironolactone reached significance on cardiovascular mortality but was not significant for MACE. CA125 (continuous) showed a statistical trend on mortality with *P* = 0.053, and predicted the combined endpoint of death/HF rehospitalization with *P* = 0.002. No impact of LVEF (categorized), serum creatinine (categorized), and systolic blood pressure (continuous) on the endpoints was observed. Predictive values of all other variables were similar for the primary and all secondary endpoints.

**Table 3:** Univariate Cox regression for baseline variables

Variables	All-cause mortality		All-cause mortality or heart failure hospitalization	
	Hazard ratio (95% CI)	*P*	Hazard ratio (95% CI)	*P*
CA125 (/1 U/ml increase)	1.001 (1.000–1.003)	0.053	1.002 (1.001–1.004)	0.002
CA125 ≥ 65.7 U/ml	2.251 (1.520–3.332)	< 0.001	1.809 (1.315–2.489)	< 0.001
NTproBNP (/1 pg/ml increase)	1.000 (1.000–1.000)	< 0.001	1.000 (1.000–1.000)	0.021
NTproBNP ≥ 4900 pg/ml	2.414 (1.623–3.590)	< 0.001	1.505 (1.097–2.065)	0.011
LVEF (/1% decrease)	1.008 (0.995–1.020)	0.223	1.005 (0.995–1.015)	0.327
LVEF < 50%	0.804 (0.539–1.198)	0.283	0.849 (0.609–1.182)	0.332
Sodium (/1 mmol/L decrease)	0.952 (0.915–0.990)	0.015	0.988 (0.955–1.023)	0.501
Serum sodium < 135 mmol/L	2.615 (1.655–4.132)	< 0.001	1.197 (0.778–1.842)	0.414
Serum creatinine (/1 umol/L increase)	1.004 (1.000–1.008)	0.053	1.002 (0.999–1.006)	0.138
Serum creatinine ≥ 120 umol/L	1.263 (0.859–1.858)	0.235	1.150 (0.831–1.590)	0.399
Age (/1 year increase)	1.019 (0.998–1.040)	0.071	1.006 (0.997–1.016)	0.211
Age ≥ 70 years	1.705 (1.021–2.847)	0.041	1.297 (0.874–1.925)	0.197
Heart rate (/1 beat/minute increase)	1.013 (1.002–1.025)	0.021	1.006 (0.997–1.016)	0.211
Heart rate ≥ 90 beats/minute	1.464 (0.976–2.198)	0.066	1.085 (0.768–1.531)	0.644
Pleural effusion or ascites	1.795 (1.212–2.659)	0.004	1.316 (0.957–1.810)	0.091
Peripheral oedema	1.411 (0.962–2.070)	0.078	1.183 (0.863–1.623)	0.296
Etiology (ischemic)	1.316 (0.814–2.128)	0.263	1.078 (0.711–1.636)	0.724
Recent admission	2.024 (1.373–2.984)	< 0.001	1.888 (1.354–2.633)	< 0.001
Treatment with digitalis	0.643 (0.422–0.978)	0.039	0.824 (0.591–1.148)	0.252
Treatment with ACEI/ARB/ ARNI	0.692 (0.472–1.013)	0.058	0.799 (0.583–1.097)	0.166
Treatment with spironolactone	0.584 (0.337–1.011)	0.055	1.031 (0.620–1.715)	0.907
Systolic blood pressure (/1 mmHg increase)	1.001 (0.991–1.011)	0.877	1.002 (0.994–1.011)	0.610

**Table 4:** Univariate Cox regression for baseline variables

Variables	Cardiovascular mortality		MACE	
	Hazard ratio (95% CI)	*P*	Hazard ratio (95% CI)	*P*
CA125 ≥ 65.7 U/ml	2.245 (1.507–3.343)	< 0.001	1.791 (1.302–2.462)	< 0.001
NTproBNP ≥ 4900 pg/ml	2.395 (1.597–3.591)	< 0.001	1.484 (1.081–2.039)	0.015
LVEF < 50%	0.815 (0.541–1.227)	0.326	0.856 (0.615–1.192)	0.358
Serum sodium < 135 mmol/L	2.754 (1.736–4.367)	< 0.001	1.197 (0.778–1.842)	0.413
Serum creatinine ≥ 120 umol/L	1.364 (0.923–2.018)	0.120	1.145 (0.828–1.583)	0.414
Age ≥ 70 years	1.593 (0.953–2.662)	0.075	1.284 (0.865–1.905)	0.215
Heart rate ≥ 90 beats/minute	1.489 (0.985–2.251)	0.059	1.098 (0.778–1.551)	0.594
Pleural effusion or ascites	1.872 (1.249–2.805)	0.002	1.324 (0.963–1.821)	0.084
Peripheral oedema	1.390 (0.941–2.054)	0.098	1.190 (0.868–1.632)	0.281
Etiology (ischemic)	1.226 (0.763–1.969)	0.400	1.102 (0.738–1.645)	0.635
Recent admission	2.124 (1.430–3.154)	< 0.001	1.902 (1.366–2.649)	< 0.001
Treatment with digitalis	0.637 (0.413–0.980)	0.040	0.829 (0.595–1.156)	0.269
Treatment with ACEI/ARB/ ARNI	0.671 (0.454–0.991)	0.045	0.815 (0.594–1.118)	0.204
Treatment with spironolactone	0.555 (0.320–0.964)	0.037	0.999 (0.602–1.659)	0.997
Systolic blood pressure (/1 mmHg increase)	0.999 (0.989–1.010)	0.890	1.003 (0.994–1.011)	0.563

MACE, major adverse cardiovascular events

Multivariate Cox analysis using a fixed set of variables showed that CA125 was a significant independent prognostic factor for all-cause death, cardiovascular mortality, combination of death/HF rehospitalization and MACE. NTproBNP was an independent predictor for all-cause death and cardiovascular mortality, but not for the combined endpoint of death/HF rehospitalization and MACE. Other independent predictors for all-cause death were recent admission, serum sodium, and treatment with digoxin (Tables 5 and 6). No interaction was found between CA125 and NTproBNP in the final model for mortality (*P* = 0.131).

**Table 5:** Multivariate Cox models using a fixed set of baseline variables

	All-cause mortality			All-cause mortality or hospitalization for heart failure		
	Hazard Ratio	95% CI	*P*	Hazard Ratio	95% CI	*P*
CA125 ≥ 65.7 U/ml	1.879	1.191–2.965	0.007	1.688	1.126–2.530	0.011
NTproBNP ≥ 4900 pg/ml	2.129	1.415–3.205	< 0.001	1.285	0.916–1.802	0.146
Age ≥ 70 years	1.188	0.695–2.031	0.529	1.065	0.693–1.637	0.775
Serum sodium < 135 mmol/L	2.524	1.584–4.023	< 0.001	1.195	0.773–1.846	0.424
Pleural effusion or ascites	1.133	0.715–1.794	0.595	0.907	0.605–1.358	0.635
Recent admission	1.737	1.164–2.591	0.007	1.642	1.166–2.312	0.005
Treatment with digitalis	0.638	0.411–0.991	0.045	0.794	0.558–1.132	0.203

Recent hospitalization, at least 1 heart failure admission within 3 months

**Table 6:** Multivariate Cox models using a fixed set of baseline variables

	Cardiovascular mortality			MACE		
	Hazard Ratio	95% CI	*P*	Hazard Ratio	95% CI	*P*
CA125 ≥ 65.7 U/ml	1.793	1.134–2.834	0.012	1.650	1.101–2.473	0.015
NTproBNP ≥ 4900 pg/ml	2.087	1.371–3.177	0.001	1.268	0.903–1.779	0.170
Age ≥ 70 years	1.088	0.634–1.867	0.760	1.063	0.692–1.633	0.779
Serum sodium < 135 mmol/L	2.671	1.668–4.279	< 0.001	1.209	0.784–1.866	0.390
Pleural effusion or ascites	1.186	0.742–1.896	0.476	0.918	0.612–1.376	0.677
Recent admission	1.751	1.162–2.638	0.007	1.659	1.179–2.334	0.004
Treatment with digitalis	0.635	0.403–0.999	0.050	0.809	0.568–1.151	0.239

Calibration curves for 0.5 year, 1 year, and 1.5 year showed the model calibration performance was good (*P* = 0.29, 0.80 and 0.21, respectively). The area under time-dependent ROC curves for 0.5, 1 and 1.5 year were 0.790, 0.708 and 0.739, which implied that the model discrimination or separation performance was good.

## Discussion

Our study confirmed CA125's risk prediction capacity in stage D HF patients including more than half (50.6%) had LVEF > 40%. In these vulnerable subjects with advanced NYHA class III-IV, elevation of CA125 levels was strongly associated with higher risk of all-cause mortality, cardiovascular mortality, composite of death or HF readmission and MACE during follow-up, independently of NTproBNP, LVEF, age, recent admission, evidence of pleural effusion, serum sodium, and treatment with digoxin.

Stage D HF differing from other stages of HF with severe prognosis [13], has limited therapeutic options, including HT, mechanical circulatory support (MCS) or palliative therapies [12]. Accurate prognostication is a prerequisite regarding the optimal timing of advanced therapies referral, proper goals and expectations, optimal treatment strategies consistent with a patient's preferences. Unfortunately, despite broader use of prognostic tools, precise prognostication is difficult because of the different trajectory of each patient in the setting of stage D HF [12, 13]. In the last days of life, aggressive procedures are performed in many cases [14]; and previous study reported 38.0% and 45.1% referred for evaluation for advanced therapies were too sick to be eligible for MCS and HT, respectively [15].

Congestion as a strong predictor of HF-related readmission and death [16], is responsible for most of HF decompensation [17]. In the BIOSTAT-CHF study, a positive association was identified between CA125 levels and congestion surrogates including NTproBNP and a composite congestion score [9]. As a marker of congestion, CA125 was related with adverse events in acute HF patients [9, 10] and in the transition to clinical stability the role for HF surveillance confirmed [18]. In the same study above including patients with worsening HF, CA125 levels were highly predictive of mortality and combined death and HF readmission, beyond and independently of surrogates of congestion, which were replicated in a validation cohort [9]. Furthermore, elevated CA125 displayed an independent association with higher risk of long-term cardiovascular deaths/HF readmissions in subjects with mild to moderate HF [19]. The overall 1-year mortality rate was 45.0% and 1-year cumulative event (death or HF rehospitalization) rate 87.3% in our cohort in which CA125 above median was associated with 1.9-fold, 1.8-fold, 1.7-fold and 1.7-fold higher risk of all-cause death (*P* = 0.007), cardiovascular mortality (*P* = 0.012), all-cause death/HF readmission (*P* = 0.011) and MACE (*P* = 0.015) respectively after adjusting for established risk factors, when compared with subjects with CA125 below median. Thus, even in stage D HF, CA125 values can help identify high-risk groups in whom advanced therapies are urgently needed or palliative care should be considered.

According to our study, LVEF and HF aetiology were not ideal biomarkers for risk stratification in the setting of stage D HF because of no significant differences observed between subjects with CA125 above median and subjects with CA125 below median. It was reported that in the "real word" two-thirds of advanced HF patients had LVEF > 40% [20], consisting with our finding that over half had LVEF > 40%. Miñana et al. reported a positive association between higher CA125 levels with an increased risk of long-term HF-readmission burden in patients with heart failure with preserved ejection fraction (HFpEF) after an episode of HF. Similarly, we found that elevated CA125 levels were highly predictive of adverse outcomes in the setting of stage D HF in which the majority with HFpEF [21]. However, Dunlay et al. [20] reported that only 2.9% and 3% underwent HT and MCS respectively, among 936 advanced HF patients 406 (43.4%) had HFpEF; the INTERMACS profiles developed to stratify risk in advanced HF undergoing consideration of MCS, is specific for HFrEF [12]. Therefore, HFpEF needs more great attention in future.

Although CA125 was positively associated with NPs, in many cases the association is weak [9, 10, 17]. Previously noted, as a surrogate of congestion, CA125 correlated strongly and independently with inferior vena cava diameter and pleural effusion which correlated marginally with NTproBNP used as a proxy of left ventricular myocardial stretch [22]. Published studies indicated that CA125 added prognostic value to NPs, and their combination confered greater predictive capacity in acute HF [10]. Furthermore, as a parameter for right-sided HF, tricuspid regurgitation severity independently correlates with CA125 values [23]. A recent study of 2961 acute HF subjects revealed that in acute HF with right-sided dysfunction CA125 had a greater prognostic effect than NTproBNP [24]. Therefore, CA125 could be the better prognostic biomarker among subjects with right-sided dysfunction, especially in HFpEF accounting for more than 40% of advanced HF cases [20]. Considering the facts mentioned above, we envision stage D HF patients with higher CA125 values may not be eligible for MCS because of worsening right ventricular function due to increased workload and a strong association between right HF and early mortality after device placement [12, 13, 25].

Additional benefits for implementing CA125 testing in daily clinical practice arise from its standardized measurement, low cost, wide availability and stability not significantly influenced by age, gender, body weight and renal function [26, 27]. In addition, this simple and objective biomarker is better suited to general cardiologists. In stage D HF population, no single variable or prognostic score is sufficient for accurate assessment of prognosis [12, 13]. The combined use of CA125 and other factors including age, frailty, comorbidities, end-organ dysfunction, measures of functional capacity (cardiopulmonary exercise test or 6-min walk) and so on may improve risk stratification in this setting.

Our study had some limitations. First, its observational design makes it susceptible to confounding factors and bias. Second, there are no studies to validate CA125's cut-off values for diagnosis, prognosis and advanced therapies of stage D HF. Third, it is not possible to extrapolate findings to patients with severe renal dysfunction because this study included patients with baseline serum creatinine values ≤ 250 umol/L. Finally, we only evaluated the relationship between CA125 and time to the first event, in future we will focus on the association between CA125 levels and long-term recurrent HF admission.

## Conclusion

In stage D HF patients, elevated CA125 levels were highly predictive of all-cause death, cardiovascular mortality, all-cause death/HF readmission and MACE, independently of NTproBNP and other clinical risk factors, which can be used for better risk stratification. Hence, this glycoprotein should be considered as a complement for optimal risk prediction. The underlying mechanisms of CA125 in stage D HF syndromes remain unclear and more research is needed.

## Data Availability

The datasets were analysed in this study available from the corresponding author on reasonable request.

## Abbreviations

CA125: Carbohydrate antigen 125
HF: Heart failure
LVEF: Left ventricular ejection fraction
NPs: Natriuretic peptides
HT: Heart transplantation
NYHA: Advanced New York heart association
NTproBNP: N-terminal pro-B-type natriuretic peptide
MCS: Mechanical circulatory support
HFpEF: Heart failure with preserved ejection fraction
